# Understanding Esports-related Betting and Gambling: A Systematic Review of the Literature

**DOI:** 10.1007/s10899-023-10256-5

**Published:** 2023-09-22

**Authors:** Harshdeep S. Mangat, Mark D Griffiths, Shu M. Yu, Katalin Felvinczi, Ronald K. Ngetich, Zsolt Demetrovics, Andrea Czakó

**Affiliations:** 1 Centre of Excellence in Responsible Gaming, University of Gibraltar, Europa Point Campus, Gibraltar, GX11 1AA Gibraltar; 2 International Gaming Research Unit, Psychology Department, Nottingham Trent University, Nottingham, UK; 3 Institute of Psychology, ELTE Eötvös Loránd University, Budapest, Hungary

**Keywords:** Esports betting, Esports, Problem gambling, Skin betting, Adolescent gambling, Systematic review

## Abstract

Esports gambling has steadily grown in popularity alongside esports itself. While research has been increasing in the field of esports-related gambling, no study has yet reviewed the relevant literature on esports gambling. The present study aimed to comprehensively review all empirical research conducted in the wider field of esports gambling. A systematic review following the Preferred Reporting Items for Systematic Reviews and Meta-Analyses (PRISMA) guidelines was undertaken using *PsycINFO, PubMed, Scopus*, and *Web of Science* databases. Only empirical studies were included and were also assessed for potential biases using the ROBUST guidelines. A total of 30 studies from eight countries were included in the review. Esports gamblers were found more likely to be young males, likely to score high on problematic gambling scales, and likely to belong to households speaking a non-English language at home in English speaking countries. Esports gamblers are a unique type of gambling population, with rare characteristics and behaviors compared to other types of gamblers. Given the limited number of studies, there is a need for further research in this field to understand these populations, as well as the need for longitudinal research.

## Introduction

Electronic sports, more popularly known as esports, can be defined as competitive video gaming (Hamari & Sjöblom, 2017). Esports have become more technical and strategic over the past two decades, having entered the sports industry as competitions of mental and physical skill, just like other traditional sports (Kim et al., 2020). Videogames are not seen as just recreational hobbies anymore, and there are limited-yet-potential career paths in the field of gaming, that did not exist in the same capacity at the turn of the millennium. Popper (2013) noticed how *Twitch.tv* (*Amazon*’s video streaming platform) had grown widely as a medium to consume esports and videogame livestreams, and that it had established itself as the market leader in video game spectatorship. Even today, a report published by Stream Hatchet (2022) stated that *Twitch.tv* has a market share of 72% in videogame-related streaming. Some of the popular esports titles are *League of Legends (LoL), Counter Strike: Global Offensive (CSGO), Defence of the Ancients 2 (DOTA 2), Tom Clancy’s Rainbow Six Siege (R6), Fortnite*, and the *FIFA series*. As the esports industry has grown, esports spectatorship has grown with it. The *League of Legends World Championship* in 2021 peaked at just over four million viewers (excluding Chinese viewership), while the *Free Fire World Series 2021* (mobile esports) peaked at over 5.4 m viewers (Borisov, 2021; Daniels, 2021).

With this rising popularity and the additional competitive nature of esports, gambling companies have naturally explored the possibilities of offering bets and odds on the outcome of these games, and esports betting has been a regular offering by many online gambling providers (Griffiths, 2017). Sylvester and Rennie (2017) claimed the only difference between traditional sports and esports was that there is a *“lack of coherent regulation and a clearly identifiable governing body”* (Sylvester & Rennie, 2017; p. 629). Therefore, just as traditional sports betting has been an important feature of most traditional sports, the existence of esports betting is no different. Esports betting can be facilitated through multiple means and payment options. Most individuals might choose to gamble with real currency, some might choose to use virtual currencies like videogame skins (for example, *CSGO skins* or *FIFA coins*), and some users might choose to use cryptocurrencies such as *Bitcoin* and *Ethereum* (if the provider accepts them as payment).

Historically, esports betting has been associated with unregulated and ‘shady’ websites due to its association with skin betting (Griffiths, 2017; Melbourne & Campbell, 2015). Companies like *CSGO Lotto* and *CSGO Wild* were big providers of these skin gambling services, without any restrictions on minors, before being shut down because of a crackdown, as well as ‘cease and desist’ letters by *Valve* (*CSGO*’s parent company) when it sparked public interest in 2016 (Frank, 2016; Holden & Ehrlich, 2017). Esports gambling with skins has also been a grey area when it comes to legislation because videogame companies have been able to argue that skins have no extrinsic value (i.e., no value outside of the videogames they exist in), and that they cannot be legitimately cashed out for real money either (Martinelli, 2017). However, this is not entirely true because third-party unregulated skin gambling websites circumvent this by offering individuals the option to cash out their skins or winnings into other online wallets like *PayPal* (Sarkar, 2016). However, even if the option to cash-out did not exist, the gambling nature of tradable esports skins is evident in the activity of skin betting itself.

The majority of the existing early literature available on esports was not associated with esports-related gambling, but focused on esports in general, examining the comparison of esports to traditional sports, interdisciplinary connections, and the standing of esports in policy and governance. However, in recent years, there has been increased research on other aspects of esports consumption, including (but not limited to) esports-related gambling, problematic esports consumption, motivations to play esports, and esports consumers’ behaviors (Yamanaka et al., 2021). Existing systematic reviews have primarily focused on esports players’ motivations and behavior and esports spectatorship, as well as focusing on problematic video gaming, loot box buying and its relationship with problematic gambling (Bányai et al., 2019; Delfabbro & King, 2020; Pedraza-Ramirez et al. 2020). Other existing reviews on esports have focused on the current state of academic research in the field of esports (Reitman et al. 2020), the impact of esports on youth skill development (Zhong et al., 2022), or on defining an esports player for future standardization (Mendoza et al., 2023). However, no previous review has systematically reviewed the psychological literature on the behavior of esports bettors and esports-related gamblers. Therefore, the primary aim of the present review was to address this gap.

Previous research has shown there is a possibility of heavy involvement in esports gambling among adolescents and emerging adults (Denoo et al., 2021; Macey et al. 2021b; Rossi et al., 2021). Research into behavioral addictions has consistently shown that adolescents and emerging adults tend to be at higher risk of developing addictive behaviors than older adults, including both problematic gambling (Calado et al., 2017) and problematic videogame playing (Stevens et al. 2021). Ferris and Wynne (2001) noted that problem gambling was a *“behavior that creates negative social consequences for the gambler, others in his or her social network, or for the community”* (p.8). It can be argued that protecting esports consumers from gambling-related harm, especially the most vulnerable, may help in preventing future problems, including, but not limited to problematic gambling and problematic esports consumption (Czakó, 2023). In the present review, gambling-related harm is *“any initial or exacerbated adverse consequence due to an engagement with gambling that leads to a decrement to the health or wellbeing of an individual, family unit, community, or population”* (Langham et al., 2015, p.4).

The goals of the present study were to review the demographic and general characteristics of esports gamblers, the relationship of esports gambling with esports spectatorship and with videogame consumption, and the motivations underlying esports and skin gambling consumption. The study also aimed to identify gaps in the extant literature. Anybody who gambled on esports or videogame outcomes, through any form (betting, crash betting, esports-themed traditional gambling types, skin betting, etc.) was deemed to be an esports gambler and any of these activities were considered as esports gambling. The term, ‘consumption’, throughout this review has been used in the context of consumption from a consumer perspective. For example, esports consumption refers to an individual playing or watching esports in any capacity.

## Methodology

### Inclusion and Exclusion Criteria

Any study that focused on esports gambling, with primary empirical data, and was published in a peer-reviewed English language journal was included. Studies that were published in non-English language journals were still included in the screening process if they had English translated titles and/or abstracts. Studies were excluded if they were irrelevant to esports betting or esports-related gambling (such as studies related to esports but without a focus or any research regarding esports-related gambling on betting), were commentary or opinion pieces, or did not contain any primary empirical data.

### Search Strategy

**Fig. 1:**
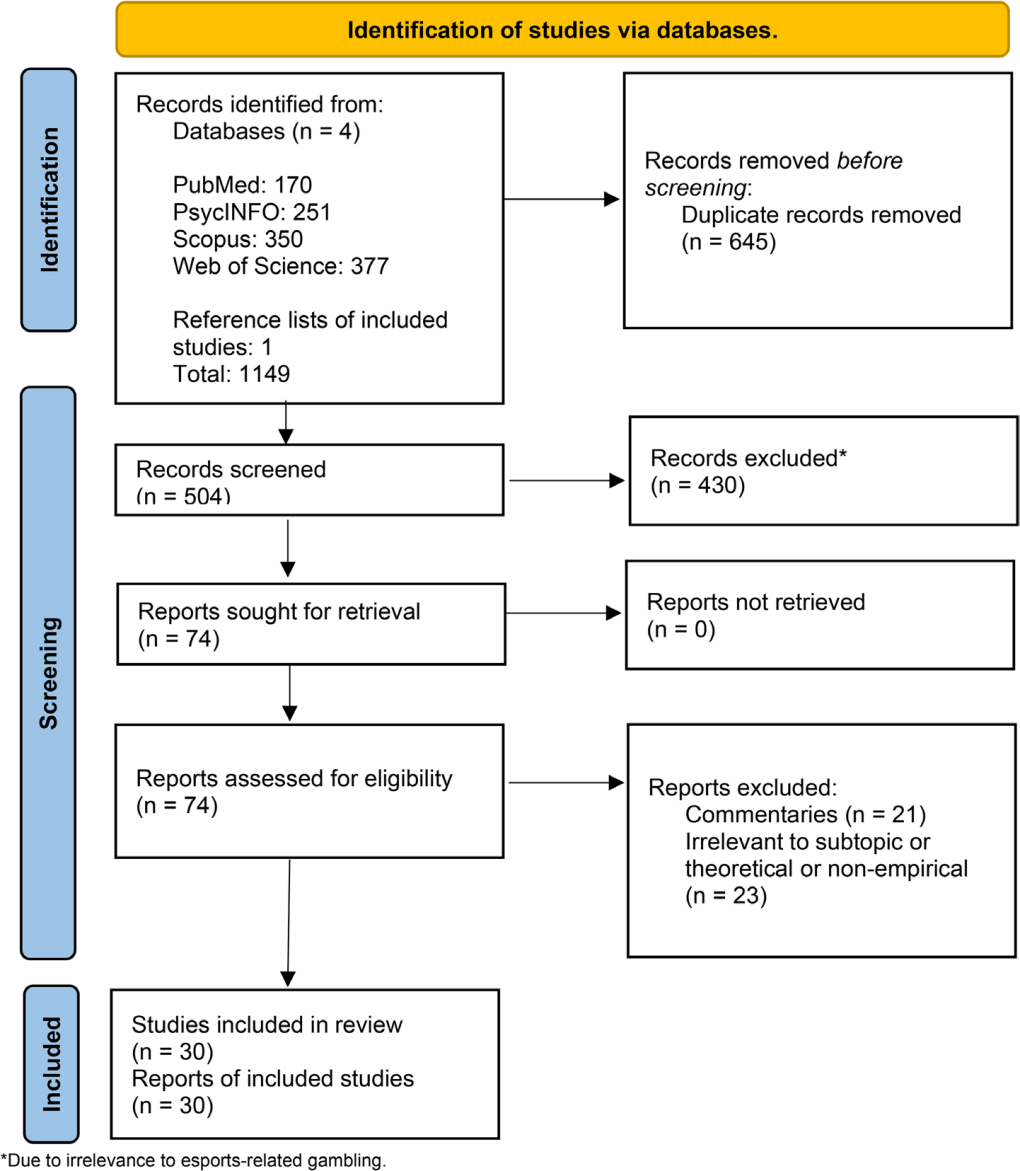
PRISMA flow chart for the search strategy and screening process (Page et al., 2021)

The research strategy (see Fig. 1) was based on the Preferred Reporting Items for Systematic Reviews and Meta-Analyses (PRISMA) guidelines (Page et al., 2021). Four databases (*PubMed, PsycINFO, Scopus* and *Web of Science*) were searched to ensure a wide variety of results. Titles, abstracts, and keywords were searched with the terms “Esport* OR E-Sport* OR Electronic Sport* OR Electronic Gaming OR Competitive Gaming” combined with “Gambl* OR Betting* OR Bettor*”. The searches had no apparent limitations outside of the English language filter. No metrics were set for date and time. The search was carried out on 21st July 2023.

The first search yielded 1148 papers, of which 645 were duplicates. Then, the remaining 503 papers were screened for relevance through the titles, abstracts, and keywords. After the initial screening, 430 papers were excluded due to being clearly irrelevant to the study (i.e., studies not focused on or esports-related gambling at all). A total of 73 relevant papers were then read in the full-text screening phase and data extraction began. During the full-text screening, all reference lists were read to identify any other studies that might be relevant, and one study was identified that met the inclusion criteria. Title, abstract and keywords screenings were completed by RN, SY and HM and any disagreements were resolved after group discussions among the three researchers and ZD. The search strategy and inclusion/exclusion criteria were also discussed and finalized by SY, ZD, AC and HM. After the final screening, 30 studies were included in the review.

## Results

### Study Characteristics

Despite the popularity of esports and the increasing emergence of the large volume of papers regarding esports over the past five years (Yamanaka et al., 2021), only 30 studies were identified that had collected empirical data concerning esports gamblers. Of the 30 studies, nine were from the USA, seven were from Australia, four were international studies, three were from the UK, two were from Finland and Poland, and there was one each from Russia, Belgium, and Turkey. Of the 30 studies, 25 were quantitative, four were qualitative, and one was mixed methods. All the included studies were cross-sectional.

### Risk of Bias

The risk of bias was assessed using the ROBUST guidelines (Nudelman & Otto, 2020). ROBUST was developed to assess the risk of bias in survey studies and each criterion uses nine-point scoring with each study graded between 0 and 8 (one point for each criterion successfully met) with 0 indicating the lowest quality (i.e., highest risk of bias) and 8 indicating the highest quality (i.e., lowest risk of bias). Four studies (Dagaev & Stoyan, 2020; Rossi et al., 2021; Russell et al., 2023; Sweeney et al., 2021) were not assessed with the checklist as they were not survey-based studies. The risk of bias assessment was carried out by three of the co-authors independently. Each study was assessed separately based on the guidelines, and then discussed in a series of meetings to ensure maximum reliability and congruency among the scorers. The final scores ranged from 3 to 7 (Table 1) for all survey-based studies, and between 2 and 4 for interview-base studies. However, it is important to note that some checklist criteria were not applicable to studies that were qualitative or mixed-methods.

**Table 1:** Risk of bias assessment of included studies

Study		1	2	3	4	5	6	7	8	Total score
		Sampling frame	Participant recruitment	Exclusion rate	Sample size	Sample characteristics	Measurement validity	Setting	Data management	
1	Abarbanel and Phung, 2019	0	0	1	1	1	N/A	0	1	**4/7**
2	Abarbanel and Johnson, 2019	0	0	1	1	0	N/A	0	1	**3/7**
3	Abarbanel et al., 2020	0	0	1	1	1	0	0	1	**4**
4	Dagaev and Stoyan, 2020	N/A	N/A	N/A	N/A	N/A	N/A	N/A	N/A	**N/A**
5	Denoo et al., 2021	0	0	1	1	1	N/A	1	N/A	**4/6**
6	Freitas et al., 2021	0	0	1	1	1	0	0	1	**4**
7	Gainsbury et al., 2017a	0	0	1	1	1	1	0	1	**5**
8	Gainsbury et al., 2017b	0	0	1	1	1	N/A	0	1	**4/7**
9	Greer et al., 2021	0	0	1	1	1	0	0	1	**4**
10	Greer et al., 2023a	0	0	0	1	1	0	0	1	**3**
11	Greer et al., 2023b	0	0	0	1	1	0	0	1	**3**
12	Hing et al., 2021	0	0	0	1	1	1	0	1	**4**
13	Hing et al., 2022a	0	0	0	1	1	1	0	1	**4**
14	Hing et al., 2022b	0	0	0	1	1	N/A	0	N/A	**2/6**
15	Lelonek-Kuleta and Bartczuk, 2021	0	0	1	1	1	1	0	1	**5**
16	Macey et al., 2021a	0	0	0	1	1	1	0	1	**4**
17	Macey et al., 2021b	0	0	1	1	1	N/A	1	1	**5/7**
18	Macey and Hamari, 2018a	0	0	0	1	1	1	0	1	**4**
19	Macey and Hamari, 2018b	0	0	0	1	1	1	0	1	**4**
20	Marchica et al., 2021	0	1	1	1	1	0	0	1	**5**
21	Nosal and Lopez-Gonzalez, 2021	0	0	0	1	1	1	0	0	**3**
22	Peter et al., 2019	0	0	0	1	1	1	0	1	**4**
23	Richard et al., 2021	0	1	1	1	1	1	1	1	**7**
24	Richard et al., 2023	0	1	1	1	1	1	1	1	**7**
25	Rossi et al., 2021	N/A	N/A	N/A	N/A	N/A	N/A	N/A	N/A	**N/A**
26	Russell et al., 2023	N/A	N/A	N/A	N/A	N/A	N/A	N/A	N/A	**N/A**
27	Sweeney et al., 2021	N/A	N/A	N/A	N/A	N/A	N/A	N/A	N/A	**N/A**
28	Wardle et al., 2020	0	1	1	1	1	0	0	0	**4**
29	Yüce et al., 2023	0	0	1	1	1	0	0	N/A	**3/7**
30	Zendle, 2020	0	0	1	1	1	1	0	1	**5**

Of the 30 studies, 24 studies were survey-based studies, two were qualitative interview studies, two studies analyzed computerized data from gambling companies on esports betting, and two studies analyzed *Twitter* advertisements.

Six studies attempted to profile esports gambling and to understand their characteristics. Hing et al. (2021) examined the characteristics of adolescent skin gamblers, their engagement in monetary gambling, and relationships between skin gambling and at-risk gambling. Gainsbury et al. (2017b) and Wardle et al. (2020) both classified and compared the characteristics of esports bettors with regular sports bettors. Richard et al. (2021) studied demographic and clinical characteristics of youth esports bettors. Richard et al. (2023) also conducted a latent class analysis among high-school students to identify sub-groups of esports bettors based on self-report measures from gaming, gambling, and loot box buying. Hing et al. (2022a) studied two samples of adolescents (n = 841; n = 826) aged 12–17 years, to explore multiple factors, including examining demographic, psychological, and videogame characteristics of esports cash and skin bettors. They also examined adolescents’ participation in other forms of gambling, variance in problem gambling severity, and the association of esports cash and skin betting on problem gambling severity while controlling for engagement in other monetary gambling forms.

Six studies investigated esports gambling behavior, and its associated harms to the participants. Gainsbury et al. (2017a) examined gambling harms and intensity among esports bettors, whereas Greer et al. (2021) studied the same, but in comparison with traditional sports bettors. Greer et al. (2023a) then analyzed a conceptual framework model linking video game involvement, videogame-related gambling, traditional gambling, and gambling problems and harm. Freitas et al. (2021) examined risk factors and/or threats present to esports sponsors’ brand image(s) from the point of view of esports fans. Peter et al. (2019) examined the public stigma attached to casino gambling, esports gambling, and internet gaming when compared to individuals facing a heavy financial crisis. Macey and Hamari (2018a) examined the harms associated with emerging forms of gambling like esports gambling, skin gambling, and videogame loot box buying.

Six studies extensively examined the relationship between other addictive behaviors and esports. Abarbanel et al. (2020) looked at the potential relationship between video gaming, esports spectatorship and sportsbook-style esports event related betting. Marchica et al. (2021) examined the relationship between esports betting, problem gambling, and problem videogame playing whereas Macey and Hamari (2018b) examined the relationship between playing videogames, watching esports, and gambling. Nosal and Lopez-Gonzalez (2021) examined esports betting as a substitute activity for sports betting during the COVID-19 pandemic when traditional sporting events came to an unexpected halt around the world. Zendle (2020) examined the associations between esports betting, token wagering, social casino spending, and real-money playing videogames. Yüce et al. (2023) interviewed sports betting tipsters (defined as individuals who provide professional tips for betting on sports) regarding their views on sports and esports betting as a substitute activity in the context of the COVID-19 pandemic.

Three studies investigated gambling predictors. Macey et al. (2021a) examined esports betting predictors over a range of behaviors associated with esports, and Macey et al. (2021b) examined esports spectatorship as a predictor of involvement in esports-related gambling. Lelonek-Kuleta and Bartczuk (2021) examined a predictive model of gambling disorder among esports bettors in addition to studying gambling motivations and coping strategies.

Abarbanel and Johnson (2019) examined esports consumers’ perspectives on match-fixing and its implications for gambling awareness and game integrity. Denoo et al. (2021) exclusively examined the motivations of esports gamblers through a series of interviews using Reynolds and Gutman’s (1988) laddering technique and the means-end chain theory. Laddering refers to a one-on-one semi-structured interviewing technique consisting of repeated and paraphrased ‘why-questions’ which encourage the participants to describe specific attributes of specific products and why they are important to the participants. In this study, the authors used the technique to identify participants’ emotions to gamble on esports using skins and real money. Greer et al. (2023b) studied the motivations for esports betting, and skin-gambling, based on seven potential factors (social, financial, positive feelings or enhancement, skill development, competition/challenge, regulating internal states, and acquiring virtual items). Finally, Hing et al. (2022b) interviewed young adults and examined their use of smartphones (compared to using computers or physical betting venues) for betting on sports, esports, and fantasy sports behavior.

Three studies examined esports gambling adverts. More specifically, Rossi et al. (2021) analyzed *Twitter* adverts with regards to volume, content, followers, engagement, and compliance to regulations, and Russell et al. (2023) analyzed *Twitter* adverts to see how four major wagering operators (*BetEasy, Ladbrokes, Sportsbet* and *TAB*), advertised and promoted their services during and around the first COVID-19 lockdown in Australia. Abarbanel and Phung (2019) studied gamers’ perception of esports gambling adverts.

Finally, two studies were data analytics studies with Sweeney et al. (2021) analyzing the market structure of the esports gambling market while Dagaev and Stoyan (2020) analyzed parimutuel betting on esports matches.

In the remainder of this section, the findings of the 30 included studies have been divided into four major topics based on the data extracted from them: (i) demographic and general characteristics of esports gamblers, (ii) esports gambling and skin gambling motivations, (iii) esports gambling and its relationship with esports spectatorship and playing videogames, and (iv) problem gambling and associated harms with esports-related gambling behaviors. An additional fifth section was also included comprising unique and idiosyncratic studies which did not directly fit in any of the four main topics but were within the scope of the review. The data extracted in this section were then further classified into subtopics.

### Demographic and General Characteristics of Esports Gamblers

Information about the general and demographic characteristics of esports gamblers were retrieved from 18 studies. Five studies (Gainsbury et al., 2017b; Hing et al., 2021, 2022a; Richard et al., 2021; Wardle et al. 2020), found that esports gamblers were more likely to come from non-white ethnic backgrounds compared to other traditional gamblers (the majority of which were sports bettors). Hing et al. (2022a) found that people who identified as Aboriginal or Torres Strait Islanders in both their samples were more likely to engage in esports cash betting compared to other groups. All five studies also noted that esports gamblers are more likely to gamble on a greater number of gambling activities, like sport betting and betting money on other games of skill, and more frequently than other traditional gamblers. Hing et al. (2022a) also noted that their esports bettors were more likely to have engaged in lottery games, bingo, and informal betting types.

Fourteen studies (Abarbanel et al. 2020; Denoo et al., 2021; Greer et al., 2023a, b; Hing et al., 2022a, b; Lelonek-Kuleta & Bartczuk, 2021; Macey et al. 2021b; Macey & Hamari, 2018a; Nosal & Lopez-Gonzalez, 2021; Richard et al., 2021, 2023; Wardle et al. 2020; Yüce et al., 2023) reported that there was a male majority in their esports betting and/or skin gambling samples (ranging from over 60%–100%). Three studies (Gainsbury et al., 2017b; Greer et al., 2021; Hing et al., 2021) did not explicitly state this, but had an overall male majority of participants (ranging from 55.1% to 71.2%). Similarly, Rossi et al. (2021) conducted a data analysis of esports gambling advertisements on *Twitter* and found that 89% of these posts had pictures of men, indicating a seemingly deliberate effort by the gambling companies to further promote esports betting to male users.

Five studies (Abarbanel et al. 2020; Gainsbury et al., 2017b; Greer et al., 2021; Macey et al. 2021b; Nosal & Lopez-Gonzalez, 2021) also noted that their esports bettor samples showed their participants to be younger in comparison to other forms of gamblers in their comparison groups. Abarbanel et al. (2020) noted that those who bet on esports (n = 140) were statistically younger than those who did not bet on esports (n = 1188). In fact, Macey et al. (2021b) (n = 255) found that 60% of their weekly and monthly gamblers (related to esports betting) were under the age of 18 years, and 50% of their daily gamblers were under the age of 18 years, while only 20% of their participants who reported gambling associated with esports and videogames were older than 25 years. Denoo et al. (2021) in their laddering interviews suggested that underage gamblers might be drawn into gambling through the unregulated and easy to bypass restrictions of ID checks or credit cards, on some esports gambling websites (through skin gambling). This was also apparent among the adolescent sample of Hing et al. (2022a) who reported esports skin betting to be more popular than esports cash betting. Additionally, Rossi et al. (2021) reported that child follower engagement in esports gambling advertising (28%) was much higher compared to that of traditional gambling advertisements (5%), with engagement slowing down after the age of 24 years. Engagement on *Twitter* was indicated by any interaction with the tweet in the form of retweet, quote retweet, reply or a like. Gainsbury et al. (2017b) and Greer et al. (2021) both found that esports betting had a higher proportion of females compared to sports betting, whereas Richard et al. (2021) found the opposite, albeit with a very small effect size. However, Richard et al. (2021) only had 25 esports bettors in their sample (8.4%).

### Esports Gambling and Skin Gambling Motivations

Four studies investigated the motivations related to esports betting and participation. In interviews (n = 13), Denoo et al. (2021) reported that participants who preferred esports betting with real money rather than skins felt that they preferred to be in control of their finances, and that the extrinsic and perceived value of skins used to gamble with were too volatile to be considered reliable. They also felt skin betting was less efficient, and just a slower way of achieving the same results and returns as they would have had if they bet real money instead. They also reported that feeling they won money by relying on skill and knowledge about esports in betting increased their self-esteem. Individuals also said they felt a bigger sensation of rush and excitement when real money was involved. Moreover, participants that preferred using skins to gamble on esports reported skin gambling was easier to control and resist tendencies to gamble because of emotional attachments to the skins. Also, gambling with skins that they earned or got for free were bonuses and could feel the difference between them and skins they purchased with real money. They did not feel any emotional or strong connections to the skins they did not like, or had duplicates of, meaning parting with them for gambling was treated as free tokens to wager for some individuals. Skins also allowed them to visualize their profits, while always ensuring that every win from a skin gambling yielded another skin that served a purpose outside of its market monetary value. A few participants were quick to point out how skin gambling enabled them to gamble on unregulated websites through just skins, without any formal identity checks and acted as gateways for them to gamble before they were 18 years.

Greer et al. (2023b) studied motivations for esports cash and skin betting, and skin gambling among adults (n = 736) to see if motivations were different based on activity/product, and associations between these motivations and gambling frequency, harms and problems. For esports cash bettors, the primary motivations were financial (to win money), and enhancement (e.g., positive feelings of excitement, thrill, fun, entertainment, and enjoyment). The secondary motive was competition/challenge. For esports skin bettors, the primary motives were skin acquisition (collection, exchange or use of skins in videogames), enhancement, and financial motives. Similarly, skin gambling (on other games of chance) was motivated by skin acquisition, enhancement, and financial motives. Social factors, skill building, and regulation of internal states were less important motives for all these groups. They found that skill building was the only significant predictor for esports cash betting while regulation of internal states and competition/challenge were significant in predicting esports skin betting. However, skin gambling was predicted by age (being younger) and competition/challenge. Overall, engagement in these activities were all driven primarily by amplified positive feelings such as excitement or winning money in cash or skins.

Lelonek-Kuleta and Bartczuk (2021) reported that escape coping strategies, the effect of paying money for progression in pay to win (P2W) games, and financial motivations were predictors for gambling disorder among esports bettors (n = 438). Macey et al. (2021a) used an adapted version of the Motivation Scale for Sports Consumption (MSSC) (Trail, 2012; Trail & James, 2001) among esports spectators. They found the consumption of esports had a positive relationship with esports betting and using esports betting websites. However, they also acknowledged that the MSSC motivations’ predictive power for esports consumption had only small significant relationships with esports betting activities.

### Esports Gambling and its Relationship with Esports Spectatorship and Playing Videogames

Eleven studies examined the potential relationships between esports gambling, esports spectatorship, and playing videogames. Seven studies (Abarbanel et al. 2020; Hing et al., 2022a; Macey et al., 2021a, 2021b; Macey & Hamari, 2018b; Marchica et al., 2021; Richard et al., 2021) reported positive associations between esports consumption, playing videogames, and esports betting. Frequency of playing videogames/esports consumption were found to be significant predictors of esports betting. Marchica et al. (2021) and Macey and Hamari (2018b) both found a positive relationship between playing videogames habits and esports consumption, with the latter study also reporting a positive relationship between problematic videogame playing and esports betting. Additionally, 88.6% of esports bettors (n = 438) from Lelonek-Kuleta and Bartczuk’s (2021) sample also played free online videogames. 67.4% of their esports bettors also spent real money to purchase add-ons in these videogames.

Six studies (Abarbanel et al. 2020; Greer et al., 2023a; Hing et al., 2022a; Macey et al. 2021b; Macey & Hamari, 2018a, 2018b) reported a positive relationship between esports spectatorship and esports betting. Three of these found esports viewing (i.e., consumption frequency of watching esports) as being a statistically significant predictor of esports-related online gambling. Most of Abarbanel et al.’s (2020) esports bettors (90%) had previously watched an esports event while Macey and Hamari (2018a) reported that only 7.4% of their esports gamblers were not esports viewers. Abarbanel and Phung (2019) found that esports bettors and esport spectators were more likely to have recalled seeing gambling advertisements in esports than non-esport spectators and non-gamblers. These groups also considered these adverts as appropriate or somewhat appropriate compared to non-gamblers and non-spectators.

### Problem Gambling and Associated Harms with Esports-related Gambling Behaviors

Thirteen studies examined problem gambling severity of their samples, or the harms associated with esports gambling. Eight of these studies (Gainsbury et al., 2017a; Greer et al., 2021, 2023a; Lelonek-Kuleta & Bartczuk, 2021; Macey & Hamari, 2018a; Richard et al., 2021, 2023; Wardle et al., 2020) noted that their esports gamblers scored significantly higher than their comparison groups (sports bettors, internet gamblers, non-gamblers, other gamblers, and non-bettors) or expected scores on problem gambling scales. Greer et al. (2023a) found a strong positive association between scores on the Problem Gambling Severity Index (PGSI) (Ferris & Wynne, 2001) and the Short Gambling Harm Screen (SGHS) (Browne et al., 2018) in their samples. Greer et al. (2021) reported that 81.9% of esports bettors (n = 298) were identified as being harmed from problematic gambling on the SGHS, compared to 45.3% for their sample of sports bettors (n = 300). Also, the average number of harms experienced from gambling for the esports bettors (mean = 4.30) were also higher than the average number of harms experienced from gambling on sports (mean = 1.93). The mean PGSI scores of esports bettors for two of these samples (Gainsbury et al., 2017a; Greer et al., 2021) were very high (9.64 for 160 participants, 10.03 for 298 participants, respectively). Moreover, 53% of esports bettors (n = 104) in Wardle et al.’s (2020) sample had a PGSI score of above 8 indicating high problematic gambling severity. Zendle (2020) also reported a positive association between esports betting and problem gambling.

Hing et al. (2022a) also found a significant positive relationship between at-risk and problem gambling with recent engagement in esports skin betting for both their samples, and for esports cash betting in one of their samples. Individuals suffering from problematic or at-risk gambling were more likely to have participated in esports skin betting, even when recent esports cash betting was controlled for. Results also indicated that individuals in the esports skin betting sample were three times more likely to be at-risk or suffer from problematic gambling. Similarly, Greer et al. (2023b) also found that the financial motivation for esports skin betting and skin gambling was significant in predicting higher-at-risk gambling category, but not for esports cash betting. In an interview study, all of Yüce et al.’s (2023) 85 sports betting tipsters (including those who bet on esports) were reported to have at least some risk of problematic sports betting, and majority of them claimed that in situations like the COVID-19 pandemic, they would bet on esports if they could not find other sporting alternatives to bet on.

Five studies (Gainsbury et al., 2017a; Greer et al., 2021, 2023a; Lelonek-Kuleta & Bartczuk, 2021; Richard et al., 2021) reported esports gambling as a major predictor of gambling on other traditional types of gambling. Wardle et al. (2020) reported individuals who spent money on loot boxes were also more likely to bet on esports, compared to those that did not. Macey and Hamari (2018a) found that most of their participants who were loot box buyers in videogames also reported participating in skin gambling. Moreover, Hing et al. (2021) reported a positive association between past month’s skin gambling and actual monetary gambling.

Richard et al. (2023) conducted a latent class analysis among high-school students (n = 5997) and identified four distinct classes of esports bettors (ESBs; n = 330) among the larger sample. Class 1 (13% ESBs) comprised individuals with the greatest risk of problematic videogaming consumption, and who scored high on gaming frequency, disordered gaming, and preference for virtual life, and exhibited a low frequency of esports betting. Class 2 (14% ESBs) comprised adolescents who had high gambling frequency in general (including different types of gambling but not esports betting), and had the highest risk of problematic gambling consumption despite having low frequency of esports betting, and lower engagement in gaming than Class 1. Class 3 (39% ESBs) comprised individuals at low risk of both gaming and gambling problems, with low frequency of esports betting engagement and having a low preference for a virtual life, and low scores on the Risky Loot Box Index (RLI, Brooks & Clark, 2019), which assesses an individuals’ risky loot box buying, problematic behaviors, and actions engaged in buying loot boxes in videogames. Class 4 (34% ESBs) comprised individuals with the highest rates of esports betting, gambling, and videogame consumption. This class had average scores on the NORC DSM-IV Screening for Gambling Problems (NODS-CLiP; Toce-Gerstein et al., 2009) and the Gaming Disorder Test (GDT; Pontes et al., 2021) suggesting elevated risk levels of problematic gaming and gambling. The NODS-CLiP assesses gambling disorder using three dichotomous yes/no questions on loss of control, lying, and preoccupation, whereas the GDT is a psychometric tool that assesses gaming disorder. However, this group also had significantly lower levels of anxiety symptoms compared to Class 1 individuals.

### Empirical Studies in Other Areas of Esports Gambling Research

Eight studies (Abarbanel & Johnson, 2019; Dagaev & Stoyan, 2020; Freitas et al. 2021; Hing et al., 2022b; Peter et al., 2019; Rossi et al., 2021; Russell et al., 2023; Sweeney et al. 2021) examined other aspects of esports-related gambling. These studies explored further aspects of esports-related gambling research and focused on specific areas that have been understudied and under-researched, when compared to the previously reported research areas. Two studies examined esports-consumer perspectives on behaviors such as match-fixing and esports gambling.

Most participants in Abarbanel and Johnson’s (2019) study (n = 1321) tended to believe that match fixing is not as serious an issue in esports as it is in traditional sports, and that it is a problem when it affects gambling and bets associated with it. They explored the impact of match-fixing on integrity as a part of their thematic analysis and found that participants felt it ruined the integrity of the game. However, some subthemes that came up discussed mitigating circumstances that lowered the severity but considered it to be acceptable at lower levels and with younger age groups of players. Reasons for these could have varied but one respondent argued that younger players are easily intimidated by older players, while another respondent felt that a bribed minor should get absolutely no punishment.

Freitas et al. (2021) reported that brands who partnered with esports entities (pro-players, organizations, teams, and tournaments) connected to illegal or unregulated gambling were not at all well perceived by esports fans (n = 1592). Their analysis reported seven disreputable behaviors in esports, that could threaten the public perception and brand image of potential esports sponsors. These were toxic behavior, sexism, illegal and unregulated gambling, match-fixing, cheating, cyberattacks, and doping. However, they only found one high-risk threat of disrepute – illegal and unregulated gambling.

Two studies examined esports gambling data from gambling providers to understand betting patterns of esports gamblers on specific videogames. Dagaev and Stoyan (2020), working with a combined database of 3075 *CSGO* esports matches demonstrated that reverse favorite-longshot bias (RFLB) exists among esports bettors in parimutuel betting in *CSGO*. RFLB refers to over-betting on favorites in a competitive match-up. The researchers theorized that if the bias is strong enough then it can lead to an inefficiency in the market because players can potentially keep extracting positive profit by playing simple strategies of betting on favorites (or vice-versa with underdogs). Their findings suggested that in matches with popular underdogs (teams that might be underdogs in a match-up but enjoy a good amount of support and fanbase, i.e., more popular than their opponent even if the opponent is a favorite), sentiment bias (a situation where the popularity of teams influences the distribution of bets) led to a decrease in market inefficiency caused by the RFLB. They found that there were strategies that can beat the market, but esports bettors tended to not exploit these strategies when popular underdogs were playing because they tend to bet on their favorite teams rather than the better team. Sweeney et al. (2021) analyzed data from professional esports matches (i.e., 2396 *Dota 2* matches, and 2775 *CSGO* matches). They reported that bettors in real money markets tended to over-bet on large underdogs (i.e. teams with subjective win probabilities less than or equal to 0.28). In esports betting, they reported evidence of favorite longshot bias (over-betting on underdogs) in the real money market but found nothing similar in the skin betting market.

Two studies analyzed esports gambling tweets from *Twitter* (now known as *X*). Rossi et al. (2021) analyzed 890,000 gambling advertisements from 417 esports gambling advertising accounts on *Twitter*. In addition to these advertisements, they analyzed data from 620,000 people who followed these *Twitter* gambling advertising accounts, with approximately 457,000 engagements. They found that esports accounts were almost twice as likely to advertise overnight between 10pm to 6am, meaning they encouraged individuals to gamble alone at inappropriate times such as late nights and early mornings. Russell et al. (2023) analyzed 53,000 *Tweets* from Australian gambling companies and categorized them into six categories, namely sports, racing, novelty, responsible gambling, other, and esports/table tennis. The authors noted that esports and table tennis were clustered together in their own category because they were usually not promoted by the gambling operators but were promoted more than usual during the lockdown period. Their analysis showed a high availability of newer forms of betting (especially esports betting) to adapt to lockdown market conditions, especially by the gambling operators *BetEasy* and *Ladbrokes*. These companies introduced promotion of esports and table tennis betting during the lockdown. However, upon the resumption of live sports, sports betting tweets took over the bulk of tweets made by these accounts, largely at the expense of esports and table tennis. On average, *BetEasy* made 3 esports/table tennis betting tweets per week just four weeks after the lockdown compared to 14.2 during the lockdown, followed by *Ladbrokes* with 0.3 vs. 9.3, *Sportsbet* with 0 vs. 1.6, and *TAB* with 0 vs. 0.2.

Peter et al. (2019) examined the social stigmatization and perception of esports gamblers among a sample of US adults (n = 504). They used four vignettes as independent variables, with all of them including a young white male called Michael. One of the vignettes portrayed Michael as going through turmoil and financial stress after securing a house loan followed by being laid off from his job. The other three vignettes portrayed him as an individual suffering from an addiction (i.e., with an addiction to either casino gambling, esports gambling or internet gaming). They found that individuals suffering from esports gambling addictions were perceived to be just as dangerous, feared, and significantly socially stigmatised as individuals suffering from casino gambling addictions, and significantly more dangerous than individuals with internet gaming addictions and individuals in a financial crisis.

In a qualitative study of interviews with 33 sports bettors, Hing et al. (2022b) included a relatively small sample of esports bettors (n = 13). Although their findings regarding esports bettors were limited they found the complexity of betting activity influenced the choice of device used for betting. Some esports bettors may have preferred using a computer because using a computer allows an individual to have multiple interfaces open at the same time. All esports bettors used smartphones to bet in some capacity, ranging from only smartphone (1/13), mainly smartphone (7/13), sometimes smartphone (3/13), and occasionally smartphone (2/13) whereas 10/13 also mentioned using a computer to bet in some capacity. Three esports bettors said they preferred using a smartphone due to having access to it all the time. However, gambling on physical venues were seen as undesirable. The authors also noted that (i) nearly all the participants mentioned that they bet more money in venues when watching events with friends than when betting alone, (ii) some participants reported how access to gambling whenever, anywhere, and everywhere can lead to problems, (iii) three participants said betting had been integrated into their daily lives, and they often used it to pass time and often checking their smartphones, (iv) two participants said privacy was important to them for betting, because it avoided stigma, and (v) two participants said they were aware that inducements influenced their betting in several ways.

## Discussion

The present systematic review of the empirical literature shows that there has been an increase in esports betting research over the past six years. Some of the included studies started off by comparing esports bettors to sports bettors (Nosal & Lopez-Gonzalez, 2021; Gainsbury et al., 2017b; Greer et al., 2021). However, the findings suggest that esports betting and sports betting do not have a lot in common between them when it comes to demographic characteristics and behavior. Esports bettors exhibited higher levels of problematic gambling compared to sports bettors and tended to be younger than sports bettors. However, these were not the only notable differences.

For example, Nosal and Lopez-Gonzalez (2021) found sports bettors preferred to bet on niche football games and unpopular sports rather than bet on esports gambling when most live sports were suspended due to COVID-19 pandemic. This is perhaps explained by their lack of understanding of esports or a higher perceived level of risk and distrust from betting on new forms they do not understand. Yüce et al. (2023) found similar preferences among their sports betting tipsters who turned to esports betting during the COVID-19 lockdown. They felt like esports betting filled the gap of real sports to some extent for bettors, but most sports bettors still believed that traditional sports betting was more exciting and better. This led to some participants to reduce their bets while eagerly waiting for traditional sports betting to resume and were adversely psychologically affected by the lack of sports betting opportunities, which led them to seek alternatives. However, it is important to note this entire sample comprised individuals suffering from potential gambling harms and may not represent the views of the typical sports bettor.

Denoo et al. (2021) concluded that esports bettors perceived that esports betting required a higher degree of skill and knowledge than traditional betting. This erroneous belief was a recurring theme among the esports bettors in Denoo et al.’s (2021) sample. Such a belief might give them a false perception regarding betting in general and make them feel like they might bet more productively on other types of betting. The review’s findings also indicated that esports gamblers exhibited higher levels and potential involvement in other forms of gambling, while showing significant relationships with esports spectatorship and playing videogames. This may be explained by the fact that esports consumption may be comparatively more popular among younger audiences. It has been widely acknowledged in existing literature that younger people have some of the highest risk levels in developing problematic gambling behavior (Calado et al., 2017) As aforementioned, the findings to date concur that esports consumers tend to be younger and score higher on problematic gambling measures. This suggests the need to push for developing measures to protect youth from gambling-related harms in relation to esports betting.

Some important gaps identified by the review suggest opportunities for future research. There is a need for the development of a dedicated measure for assessing esports consumption motivations, to ensure a clear difference in theoretical discussions regarding esports and traditional sports content (Denoo et al., 2021; Macey et al., 2021a). Secondly, future research should inform policy and regulation and explore potential harm minimization interventions for esports gamblers. Research should also focus on increasing awareness regarding the existence and potential harms of esports-based gambling to parents and young videogame players (Greer et al., 2019). Richard et al. (2023) found that not all adolescent esports bettors experience gaming, gambling or mental health problems, and this may be due to negative behaviors associated with excessive gambling not having yet reached problematic levels due to infrequent consumption at that age. This further reinforces Greer et al.’s (2023b) call for public health and education programs to discourage young people from participating in esports betting and skin gambling, before any potential problematic consumptions arise.

Peter et al. (2019) proposed that future research should focus on developing interventions targeting public stigma of gaming and gambling disorders to reduce the effect stigma has on individuals suffering from these disorders, and to help individuals recover from problems with esports gambling. Skin-based gambling should be investigated more extensively, especially its role as a virtual currency in facilitating easier esports betting (Greer et al., 2023a; Hing et al., 2022a), and (given the lack of regulation) the potential harm it poses to underage users (Denoo et al., 2021; Hing et al., 2022a; Lelonek-Kuleta & Bartczuk, 2021). There is also a lack of research in studying causal relationships between videogames and gambling-like behavior (including esports betting), and problem gambling (Zendle, 2020). Therefore, there is a need for longitudinal studies in the field to study these potential causal relationships (Marchica et al., 2021).

Overall, the volume of research is still lacking in the field of esports-related gambling. Firstly, all included studies in the present review were cross-sectional, pointing to a need for longitudinal studies and analyses in the field of esports-related gambling. There is also a lack of research studying samples of parents including their perceptions and awareness regarding esports gambling. The limited research indicates that esports gamblers appear to be comparatively younger than other gamblers, and there appears to be more underage involvement in esports gambling compared to other types of gambling (like sports betting). The findings also suggest that parents need to be more aware of the existence of competitive videogame playing and esports betting, as well as their children’s exposure to microtransactions and other gambling-like behavior in videogames and esports (Király et al., 2021). Parents need to understand that esports gambling can be facilitated by alternative forms of payment like skins and virtual currency. Research is also needed on whether restrictive parenting (referring to parental practices of developing and implying regulative rules over the media use of children) influences a child’s prospective relationship with problematic esports gambling consumption (Lukavská et al., 2022) and whether esports gamblers perceive less risk and harm from using skins (that might be gifted by somebody or purchased by their parents) compared to gambling with real money.

Research in English-speaking countries has also indicated that esports gamblers were more likely to (i) speak a non-English language at home (Greer et al., 2021) and (ii) come from non-white ethnic backgrounds (Wardle et al., 2020). While there has been relatively more research in non-English speaking regions like Scandinavia, there has hardly been any study of esports gambling in non-Caucasian regions including Asia, Central and South America, and Africa. Replication studies are needed in countries where gambling culture is informal and unorganized. For example, India is a big consumer of mobile videogames and mobile esports (Agrawal & Upadhyay, 2022) but gambling in most forms is heavily restricted (Adhikari & Misra, 2022). It is also unknown whether individuals in countries with lower per capita incomes, lower human development indexes (HDIs) and/or lower levels of development exhibit similar gambling behaviors in esports gambling compared to more developed and richer nations.

Macey et al. (2021a) raised the possibility of developing exclusive measures to assess esports consumption and esports-related gambling motivations. Future research should examine if existing gambling motivation assessment measures and scales could be adjusted to fit esports, or if there is a need for revisions. Moreover, since there have not been any special policy interventions designed for esports gambling, it is safe to assume that most governments either still have not evaluated it as a separate form of gambling, or just consider the existing policies in place to be adequate in minimising gambling-related harm. Rossi et al. (2021) made a concrete effort to propose some changes. They advocated for a range of changes with respect to problematic esports gambling advertising on *Twitter*. They also proposed amending specific gambling laws in the UK such as changing which advertisements are “of particular appeal (something that appeals more to children, compared to adults)” to what is “of a strong appeal” to children. This was proposed because their analysis found that it was not clear in 42% of their cases whether the *Twitter* esports advertising was “of particular appeal” to children or not. They also recommended using big data analytics to filter and exclude individuals under the age of 18 years, saying that social media platforms have the data available to screen these users effortlessly. Also, they proposed devising new social media-specific advertisement regulations to reduce a range of problems such as (i) exposure to esports gambling advertisements at night, (ii) accessible esports betting on smartphone apps, and (iii) the normalisation of gambling among social media users due to the increasing volume of gambling advertisements across various social media channels.

Dagaev and Stoyan (2020) and Sweeney et al. (2021) studied the behavior of esports bettors utilizing large betting datasets of esports matches. Their findings should encourage upcoming and existing researchers to investigate esports betting data from gambling companies, to identify more characteristics on esports betting behavior that might assist in developing measures to detect problematic gambling in esports bettors and facilitate efforts in harm minimization.

The present study is not without limitations. Non-English language studies were excluded from the review. Asian nations including Japan, South Korea, and China have seen a steady increase in playing videogames and esports consumption over the last few years (Niko Partners, 2022). In their report, Niko Partners (2022) stated that Asia accounts for around 57% of the global esports market. Therefore, it is possible that some important and relevant literature might have been missed from Asia due to it not being published in English-language journals. Additionally, all the studies included were arguably diverse in focus, and most (but not all) included studies were cross-sectional with most relying on self-report data. Overall, all studies were carried out in only nine countries.

## Conclusion

The present review systematically evaluated all English-language peer-reviewed empirical studies on esports-related gambling, with a key focus on esports betting. Esports betting research is increasing, but the present review shows that there is much scope for more expansive research. The review clearly highlighted the need to differentiate esports gamblers from traditional gamblers. However, future research should look towards adding more diversification to existing literature such as studying esports sponsorships and their association with esports betting (Freitas et al., 2021). Also, emerging evidence concerning motivations to gamble on esports has been promising and should act as a stimulus for future researchers to explore these further. Additionally, future research should be done with a focus on implications for policy and regulation to protect these younger age groups from potential gambling-related harms (Richard et al., 2021; Hing et al., 2021). With esports betting sponsorships being a major part of the current esports landscape, solutions to keep them safely away from repeated exposure to the underage population should be prioritized by gambling regulators and policymakers. Research should also look towards promoting healthier esports consumption habits, to avoid the possible dangers that can arise from problematic esports consumption. Empirical work has emerged from select research centres and mostly concentrated in the USA, Australia, Finland, and the UK, and even though the present review was filtered for English-only studies, and might be biased, it can be argued that on the international level, there is not a lot of research. However, future studies could investigate grey literature and studies in other languages around the world to explore this further.

## Data Availability

Not applicable.
